# Sustained Delivery of Paliperidone Palmitate via Encapsulation in Bio-Based NIPU Nanoparticles

**DOI:** 10.3390/polym18080920

**Published:** 2026-04-09

**Authors:** Maria Angeliki Ntrivala, Evangelia Balla, Ermis P. Christodoulou, Margaritis Kostoglou, Panagiotis Klonos, Apostolos Kyritsis, Dimitrios N. Bikiaris

**Affiliations:** 1 Laboratory of Polymer Chemistry and Technology, Department of Chemistry, Aristotle University of Thessaloniki, GR-541 24 Thessaloniki, Greece; ntrivalamariaangeliki@gmail.com (M.A.N.); evampadio@chem.auth.gr (E.B.); evicius@gmail.com (E.P.C.); pklonos@chem.auth.gr (P.K.); 2 Laboratory of Chemical and Environmental Technology, Department of Chemistry, Aristotle University of Thessaloniki, GR-541 24 Thessaloniki, Greece; kostoglu@chem.auth.gr; 3 Department of Physics, National Technical University of Athens, Zografou Campus, GR-157 80 Athens, Greece; akyrits@central.ntua.gr

**Keywords:** non-isocyanate polyurethane, NIPU, bio-based polymers, Paliperidone Palmitate, schizophrenia, antipsychotic, nanoemulsions, drug-delivery system, controlled release, long-acting injectables (LAIs)

## Abstract

In this study, Paliperidone Palmitate (PP), a second-generation antipsychotic, commonly used for the treatment of schizophrenia, was encapsulated in bio-based non-isocyanate polyurethane (NIPU) nanoemulsions. NIPU was synthesized via an isocyanate-free polyaddition route, addressing safety and environmental concerns associated with conventional polyurethanes. The drug-loaded nanoparticles were produced utilizing oil-in-water (O/W) emulsions followed by solvent evaporation and lyophilization. NIPU concentrations of 0.3% and 0.5% *w*/*v*, as well as 0.5% *w*/*v* PVA were employed, while PP was incorporated at 0.2%, 0.5% and 1% *w*/*v*. The formulations were characterized by FTIR, DSC and XRD analyses, and the mechanical strength of neat sponges was evaluated. The nanoparticle formation and size were assessed by DLS and SEM analyses. The water contact angle, porosity measurements and aquatic and enzymatic hydrolysis were additionally performed. The resulting nanocarriers exhibited controlled particle size, increased drug-loading values, structural stability and biodegradability. Lastly, the in vitro dissolution studies revealed a system-specific burst release behavior, and a controlled and sustained overall drug-release profile for majority of the formulations, thereby indicating the potential of NIPU nanocarriers for drug delivery applications, particularly where sustained therapeutic effects are required.

## 1. Introduction

Schizophrenia is a chronic and debilitating psychiatric disorder requiring long-term management strategies to ensure therapeutic compliance and reduce relapse risk. One of the key challenges in treating schizophrenia is medication nonadherence, which is closely linked to symptom exacerbation, increased hospitalization rates, and even suicide attempts [1]. To address this, long-acting injectable (LAI) antipsychotics have emerged as a preferred option, enabling consistent plasma drug concentrations and better adherence monitoring.

**Figure 1:**
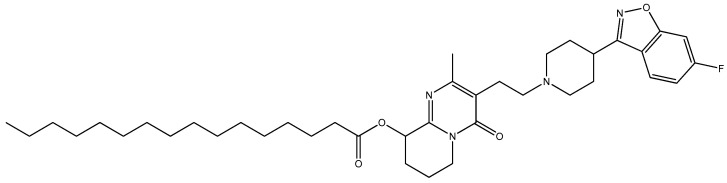
The chemical structure of Paliperidone Palmitate.

Paliperidone Palmitate (PP) (Figure 1), the palmitate ester of paliperidone (9-hydroxyrisperidone), represents a second-generation LAI antipsychotic approved for once-monthly intramuscular administration. Paliperidone, the active moiety of PP, is additionally an atypical antipsychotic that has been extensively investigated in advanced drug-delivery systems, including polymeric microspheres [2] and mesoporous carriers [3] for sustained and intranasal administration. PP functions primarily through antagonism of dopamine D_2_ and serotonin 5-HT_2_A receptors, achieving rapid therapeutic levels without requiring oral supplementation during initiation [1]. Despite its clinical efficacy, PP presents several pharmacokinetic limitations, such as poor aqueous solubility, variable bioavailability, and limited blood–brain barrier (BBB) penetration, due to its substrate status for P-glycoprotein efflux pumps [4].

In order to overcome the aforementioned barriers, the encapsulation of PP in polymeric matrices has been investigated as a promising strategy, providing sustained and controlled drug-release. Polymeric carriers such as micelles, liposomes, and micro- and nanospheres not only enhance PP’s solubility and permeability but also improve its biodistribution and pharmacokinetic profile. Representative examples of effective carrier systems that have been studied are d-alpha-tocopheryl polyethylene glycol 1000 succinate (TPGS) micelles [4], as well as biodegradable polymer-based microspheres composed of poly(alkylene succinate) polyesters [5]. TPGS micelles demonstrate the ability to encapsulate PP with high efficiency (92%) and achieve prolonged drug release exceeding 24 h, while simultaneously protecting the active pharmaceutical ingredient, enhancing BBB penetration, and reducing dosing frequency, the latter being an essential advantage in maintaining therapeutic levels for psychotic episodes [4]. Biodegradable polyesters enable tunable degradation profiles and controlled release rates, driven by polymer crystallinity, melting temperature, as well as their enhanced melt memory capability [5,6,7]. Such design flexibility enables tailored drug release kinetics to match therapeutic requirements, potentially outperforming commercial depot systems like Invega Sustenna^®^. Altogether, the encapsulation of PP in polymeric matrices either as micelles for short-term use or biodegradable microspheres for sustained release constitutes a transformative approach in antipsychotic therapy.

The selection of suitable materials and synthetic routes is a key determinant, considering safety is a crucial parameter that cannot be overlooked. Usually poly(lactic acid) (PLA) and its copolymers poly(lactic-co-glycolic acids) (PLGA) are used as appropriate drug carriers for microencapsulation [8,9,10]. Furthermore, traditional polymeric carriers, including traditional PUs, often involve toxic reagents or limited tunability, which can hinder clinical translation [11,12].

In this context, although conventional polyurethanes (PUs)—such as Pellethane^®^ [13], Tecoflex™ [14] and Carbothane™ [15]—have been extensively utilized in biomedical applications [16,17], their employment presents notable concerns regarding potential cytotoxicity and carcinogenicity attributable to products of isocyanate degradation. The synthesis of most industrially produced PUs requires the utilization of aromatic polyisocyanates like toluene diisocyanate (61.3%) and methylene diphenyl diisocyanate (34.1%), which demonstrate high reactivity and favorable mechanical properties [17]. Nevertheless, isocyanates are classified as highly toxic and carcinogenic compounds, with a documented risk of triggering inflammatory and asthmatic responses in humans [17,18]. The reagent that predominantly generates isocyanate molecules, phosgene, is also of increased toxicity [19,20] and has even been associated with mortality [19]. Additionally, the degradation of isocyanate-based PUs generates mutagenic diamines, further undermining biocompatibility and hemocompatibility [17,18]. As a consequence, in accordance with European Regulation (EC No. 1272/2008), PUs are subject to increasing restrictions under the REACH framework due to the critical health hazards they present [17].

In response to these challenges, non-isocyanate polyurethane (NIPUs) have been developed as a sustainable, biocompatible alternative of minimal toxicity with considerable potential, owing to their environmental friendliness, structural versatility, and an improved safety profile [17,18]. The aforementioned beneficial characteristics are attributable to the absence of isocyanates throughout production, the frequent incorporation of renewable resources during NIPUs’ synthesis, as well as to the preferential adoption of the polyaddition synthetic pathway involving facile and non-hazardous reactions [18,19,20,21,22]. NIPUs’ increasing presence in biomedical applications, particularly in drug-delivery systems, arises from their capacity to integrate mechanical versatility with biological safety, thereby circumventing the adverse health effects associated with conventional PUs [17].

The present study focuses on the synthesis and characterization of novel emulsion-produced nanospheres of a NIPU-based polymeric matrix and evaluates its potential as a sustained-release carrier for PP, aiming to enhance both therapeutic efficacy and sustained drug-release. The NIPU was prepared according to the methodology described in our earlier work [23]. PP was encapsulated into the synthesized NIPU through an oil-in-water (O/W) emulsification method assisted by probe sonication. The resulting drug-loaded systems were characterized by Fourier-Transform Infrared Spectroscopy (FTIR) to confirm successful encapsulation and interactions with the polymer matrix. Differential Scanning Calorimetry (DSC) and X-ray Diffraction (XRD) analysis were performed for the determination of the melting (Tm), crystallization (Tc), glass transition (Tg), temperature values and the crystallinity of the materials, respectively. Unloaded samples underwent mechanical tests, while Dynamic Light Scattering (DLS) measurements were conducted to characterize the nanoparticle size and distribution of both loaded and unloaded NIPUs. The aforementioned nanoparticles were visualized using Scanning Electron Microscopy (SEM). Additional characterizations included the evaluation of the materials’ porosity, the investigation of their hydrolytic and enzymatic degradation, as well as the analysis of their surface wettability via water contact angle measurements. High-performance liquid chromatography (HPLC) was employed to determine the drug loading, together with in vitro dissolution studies carried out to assess the release profile of Paliperidone Palmitate from the NIPU matrices. Lastly, modeling of drug release profiles was additionally performed as an attempt to fit the experimental release data and to elucidate the underlying release mechanisms through the application of established partially empirical and partially phenomenological models.

## 2. Materials and Methods

### 2.1. Materials

Adipic acid (A.A., 99%, Alfa Aesar, Haverhill, MA, USA), 4-hydroxymethyl-1,3-dioxolan-2-one (glycerol carbonate, G.C., 90%, Fluorochem, 14 Graphite Way, Hadfield, Glossop, UK), 4-((N,N′-dimethylamino)pyridine) (DMAP, 99%, Alfa Aesar, Haverhill, MA, USA), N,N′-dicyclohexylcarbodiimide (DCC, 99%, Alfa Aesar, Haverhill, MA, USA), 1,6-hexamethylenediamine (98%, Sigma-Aldrich Chemicals, St. Louis, MO, USA), dichloromethane (DCM, ≥99.8%, analytical reagent grade, Fisher, Hampton, NH, USA), hydrochloric acid (HCl, ChemLab, Zedelgem, Belgium) and trifluoroacetic acid (TFA, Thermo Scientific Chemicals, Ward Hill, MA, USA) were employed. Furthermore, polyvinyl alcohol (PVA, Mw = 33 kg/mol, produced inside the lab) was utilized for the formation of the nanoparticles. Paliperidone Palmitate (PP, ≥98%) was kindly provided by Pharmathen S.A. (Athens, Greece).

### 2.2. Synthesis

#### 2.2.1. Synthesis of Neat NIPU Nanoparticles Through Nanoemulsions

**Figure 2:**
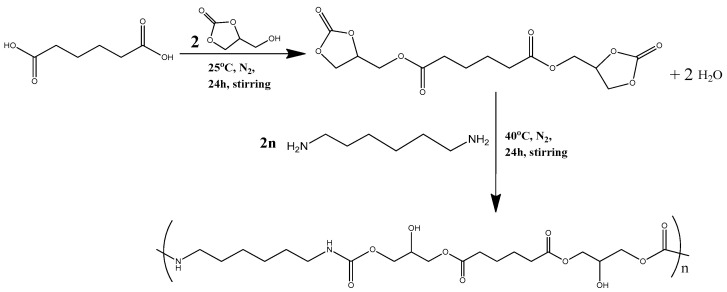
Two-step polyaddition synthetic path of adipic-acid-based NIPU, as described in our previous work [23].

NIPUS were prepared via the polyaddition route, with the employment of adipic acid, glycerol carbonate (GC) and 1,6-hexamethylenediamine, as reported in our previous work [23] (Figure 2). The production steps are also included in detail in the Supplementary Materials document, and the NMR spectra is included in Figure S1.

The synthesis of the neat NIPU nanoparticles (samples N1 and N2, Table 1) was performed through the formation and lyophilization of O/W emulsions. The power and duration of the sonication process, as well as the concentration of the emulsifier, had been optimized based on trials that had been initially performed. Accordingly, the selected and presented parameter values correspond to those producing the most stable emulsions with the smallest achievable particle sizes within the minimum possible sonication duration, as determined from the beforementioned trials. In more details, a solution of 0.5% PVA was prepared, as well as NIPU solutions with concentrations of 0.3 and 0.5% in DCM. For the complete dissolvement of NIPU, approximately 2 mL of TFA was added inside each solution. Both NIPU solutions were subsequently submerged in a bath with constant temperature conditions (37 °C) for 24 h. The emulsions were then produced in an ice bath, where 25 mL of the organic phase (NIPU solution, 0.3% or 0.5%) was added in 25 mL of the aqueous phase (PVA solution) and 10 cycles of 1 min sonication at 50% amplitude were performed (with 30 s intervals between each cycle). Following the completion of the sonication cycles, the prepared emulsions were placed under stirring at approximately 300 rpm and room temperature for ~3 h in order to achieve solvent evaporation. Thereafter, the emulsions were put inside a freezer for at least 24 h before being placed in a freeze-dryer. Stirring and freeze-drying are considered the primary processes responsible for the evaporation of the solvent; however, analytical quantification will be required in future studies to confirm its complete elimination.

#### 2.2.2. Synthesis of PP-Loaded NIPU Nanoparticles Through Nanoemulsions

The same methodology used for the neat NIPU nanocarriers (Section 2.2.1) was also employed for the preparation of the drug-loaded ones (NPP1–NPP6 samples, Table 1). The sole difference was the addition of PP to the 25 mL of the organic phase prior to the emulsion production. Specifically, for both NIPU concentrations (0.3 and 0.5%), three different drug percentages were added, 0.2, 0.5 and 1% *w*/*v*. After the addition of PP, one cycle of 1 min sonication (50% amplitude) was performed in the organic phase for ensuring complete drug dissolution. Emulsion preparation was carried out using the organic phase with the drug dissolved within it, as outlined in the preceding paragraph. Solvent evaporation was achieved through stirring (300 rpm, room temperature, ~3 h) after the completion of the emulsification process, and the final PP-loaded nanoparticles were obtained following the emulsions’ freezing and subsequent freeze-drying.

Table 1 presents all the prepared neat and drug-loaded formulations, along with their assigned nomenclature.

### 2.3. Characterization

#### 2.3.1. Characterization of Neat NIPU Nanoparticles

##### Fourier-Transform Infrared Spectroscopy (FTIR)

The determination of the synthesized materials’ chemical structures was performed through the utilization of FTIR (model FTIR-2000, Perkin Elmer, Waltham, MA, USA). The FTIR spectra of all the samples were recorded with an FTIR spectrometer, employing KBr disks, in the range 4000–400 cm^−1^ and at a resolution of 2 cm^−1^. The final spectra constituted a co-addition of 16 scans for each sample, and they were additionally normalized and baseline-corrected.

##### Differential Scanning Calorimetry (DSC)

The thermal transitions were studied by conventional DSC. The measurements were conducted employing a TA Q200 apparatus (TA Instruments, New Castle, DE, USA), properly calibrated with indium and sapphires, on samples of ~7–10 mg in mass closed in standard TA aluminum pans, in a high-purity nitrogen atmosphere. The study was conducted within the temperature range between −90 and 150 °C. Two thermal scans were performed, as follows: (scan 1) Equilibration of the sample ‘as received’ at −90 °C and heating up to 120 °C at 10 °C/min and (scan 2) the samples were cooled down to −90 °C at 20 °C/min and, subsequently, heated up to 150 °C at 10 °C/min.

##### X-Ray Diffraction Analysis (XRD)

X-ray powder diffraction (XRD) was employed for the investigation of the samples’ crystalline structure at room temperature. Diffraction patterns were collected using a Rigaku MiniFlex II X-ray diffractometer (Chalgrove, Oxford, UK) equipped with CuKα radiation (λ = 0.154 nm). Measurements were performed over a 2θ range of 5–45°.

##### Mechanical Tests

Tensile tests were performed on the lyophilized porous matrices obtained after freeze-drying using a Shimadzu EZ universal testing machine (Model EZ-LX, Kyoto, Japan) equipped with a 2 kN load cell, in accordance with ASTM D882 [24], at a crosshead speed of 5 mm/min. The specimens were prepared with the use of a hydraulic press without the employment of heat and were subsequently cut using a Wallace cutting press (Surrey, UK). Two measurements were conducted for each sample, and the reported results correspond to the average of the tensile strength at break.

##### Dynamic Light Scattering (DLS)

The nanoparticle size was determined by Dynamic Light Scattering (DLS), using a Litesizer DLS 500 instrument (Graz, Austria). A small amount of each sample was dissolved in water by vortex stirring and diluted with the same solvent at a 1:10 ratio. Measurements were performed at 25 °C, and the reported values represent the average of three scans for each sample.

##### Scanning Electron Microscopy (SEM)

Scanning Electron Microscopy (SEM) analysis was performed using a JEOL JMS-840A scanning electron microscope (Tokyo, Japan) equipped with an Oxford ISIS 300 energy-dispersive X-ray (EDX) microanalysis system (Oxford, UK). Nanoparticle samples obtained after dissolution and centrifugation of the formulations were examined, with the sediment retained and the supernatant discarded. Prior to analysis, all specimens were allowed to dry to ensure complete water evaporation and were subsequently coated with a thin layer of carbon to prevent charging effects under the electron beam.

##### Porosity Test

For the determination of the samples’ porosity, in the form of sponges, the liquid displacement method with absolute ethanol [25,26] was employed. Briefly, cubic samples of similar weight were cut, and their external volume was estimated with the use of a ruler and a digital caliper. Each sample was immersed in 10 mL of absolute ethanol for a total of 5 min. The specimens were removed and the remaining ethanol volume was calculated. The initial and final ethanol volumes (V_in_ and V_f_), as well as each sample’s external volume (V_s_) were then utilized for porosity (ε) calculation. The ethanol volume inside the samples (V_in_ − V_f_) is equal to their void/pore volume. Generally, porosity represents the percentage of a sample’s pores relative to its total volume (external and pore volume combined). Therefore, the utilized equation for ε determination is the following:

(1) $$\varepsilon =\frac{V_{pores}}{V_{total}}\times 100=\frac{\left(V_{in}-V_{f}\right)}{V_{s}+(V_{in}-V_{f})}\times 100=\frac{\left(10-V_{f}\right)}{{10+V}_{s}-V_{f}}\times 100$$

##### Hydrolysis and Enzymatic Hydrolysis

The hydrolysis of the prepared loaded and non-loaded NIPU/PVA formulations in the form of sponges took place in an aqueous PBS buffer solution (pH 7.4). For enzymatic hydrolysis, *Rhizopus arrhizus* and *Pseudomonas cepacia* lipases were also added to the PBS solution. The mean value of the materials’ hydrolysis was determined by measuring the mass losses and weights of similar-sized specimens (approximately 1 × 1 cm) that had been immersed in 10 mL of the PBS buffer and PBS–enzyme buffer. Each sample was placed in a closed jar and kept at 37 °C. The specimens were taken out every 5 days, dried under vacuum at 50 °C for 24 h, weighed on a scale and re-immersed in renewed media until a period of approximately one month (almost 30 days) was reached. Each measurement was repeated two times, and as a result, the degree of the hydrolysis and enzymatic hydrolysis of the sponges was estimated by the average sample weight loss.

#### 2.3.2. Characterization of PP-Loaded NIPU Nanoparticles

Further analysis of the PP-loaded materials included the following:

##### Water Contact Angle Analysis

The samples’ surface wettability was evaluated using an Ossila Contact Angle Goniometer (Model L2004A1, Sheffield, UK) with the sessile drop method. Thin-films of the materials were prepared with a hydraulic press to ensure a uniform surface. A 25 μL droplet of distilled water was deposited onto the sample surface, and the contact angle was recorded at t_0_ = 0 s and t_f_ = 0.95 s employing a high-resolution camera. Video recordings were analyzed with Ossila Contact Angle Software version 4.2.1 (Ossila Ltd., Sheffield, UK). Measurements were performed at least three times for each sample, and the mean value is reported.

##### Drug Loading

The nanoparticles’ drug-loading content was determined by the application of the following equation:

(2) $$Drug\;loading\;\%=\frac{weight\;of\;PP\;in\;nanoparticles}{sample\;weight}\times 100$$

Approximately 10 mg of each drug-loaded sample was dissolved in 10 mL of DCM under constant stirring for 24 h. The dissolved samples were filtered (filter diameter 0.45 μm) and analyzed through HPLC (Section High-Pressure Liquid Chromatography (HPLC)).

##### In Vitro Dissolution Studies

In vitro dissolution drug-release studies were conducted using a DIStek Dissolution Apparatus (Evolution 4300, North Brunswick, NJ, USA) equipped with an autosampler. For the drug-loaded nanoparticles, the USP dissolution apparatus I (basket method) was employed. A sample amount of approximately 10 mg was placed in dialysis cellulose membrane bags (molecular weight cut-off 12,400 Da), sealed and positioned inside the baskets. This procedure is consistent with previously reported in vitro dissolution studies of PP-loaded carriers [4,5]. The dissolution medium consisted of 500 mL phosphate-buffered saline (PBS, pH 7.4) maintained at 37 °C, with a stirring rate of 50 rpm, to promote sink conditions by using a large release medium-volume relative to the sample mass, ensuring that the drug concentration in the medium remained well below its saturation solubility during the experiment. At predetermined time intervals, approximately 1 mL was withdrawn from the release medium, filtered (filter diameter 0.45 μm) and analyzed by HPLC.

##### High-Pressure Liquid Chromatography (HPLC)

The determination of the drug content was achieved through High-Pressure Liquid Chromatography (HPLC). In more details, the Shimadzu HPLC system (model LC-20AD, Tokyo, Japan) utilized consisted of a degasser (Model DGU-20A5, Tokyo, Japan), a pump (Model LC-20AD, Tokyo, Japan), a manual injector with a 100 μL loop (Model Rheodyne, Cotati, CA, USA), a variable wavelength UV–Vis detector (Model SPD-20A, Tokyo, Japan), and a column oven (Model CTO-20AC, Tokyo, Japan). Athena C18 (CNW Technologies, Düsseldorf, Germany) 5 μm, 120 Å, 250 mm × 4.6 mm analytical column was employed. The flow rate was set at 1 mL/min and the column temperature was maintained at 25 °C. A diode array detector was used at 269 nm and the quantification of PP was based on a calibration curve prepared at various concentrations (0.005–50 ppm) of PP in the mobile phase, which consisted of water (pH 2.0)/ACN (10/90, *v*/*v*) and methanol in a final ratio of 90:10 (*v*/*v*).

## 3. Results and Discussion

### 3.1. Synthesis of the Materials

The adipic acid-based NIPU, the drug-free NIPU nanoparticles (N1, N2) and the drug-loaded ones (NPP1–NPP6) were successfully synthesized, as indicated predominantly by the results of the following characterizations, as well as the NMR spectra of the intermediate and the neat NIPU presented in the Supplementary Materials document. The molecular weight (Mw) of the neat NIPU was determined to be 7290 g/mol with a PDI of 1 through GPC analysis. For the aforementioned characterization, calibration was performed using polystyrene standards.

### 3.2. FTIR

**Figure 3:**
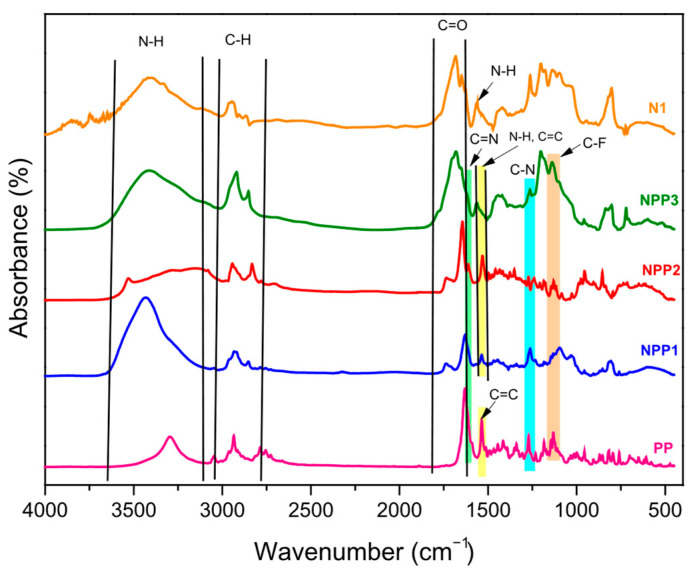
FTIR spectra of N1, PP and NPP1 to NPP3 samples.

**Figure 4:**
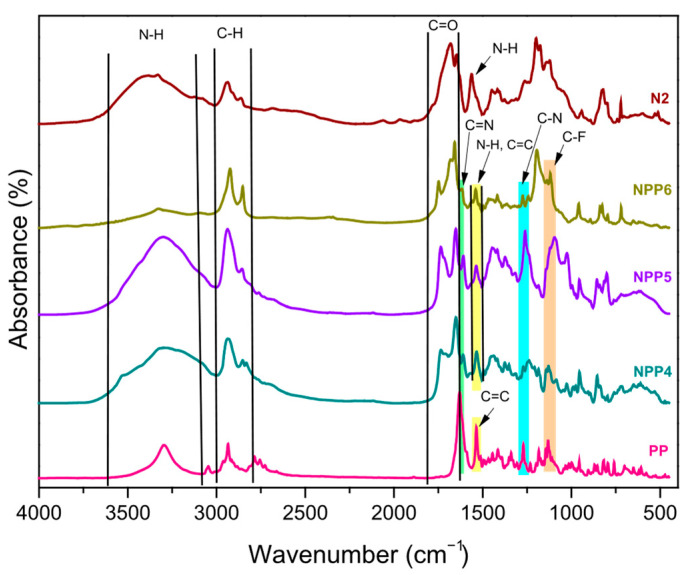
FTIR spectra of N2, PP and NPP4 to NPP6 samples.

The FTIR spectra of the neat formulations (N1, N2), the drug (PP) and the drug-loaded systems (NPP1 to NPP6) are presented in Figure 3 and Figure 4. All the characteristic peaks of Paliperidone Palmitate were detected in the drug spectrum, including peaks at 1130 cm^−1^ (C-F single bond), 1271 cm^−1^ (C-N bond), 1535 cm^−1^ (C=C double bond), 1613 cm^−1^ (stretching vibration of the C=N imine double bond), 1640 cm^−1^ (C=O bond), 2830–2990 cm^−1^ (C-H bond) and 3295 cm^−1^ (a relatively broad band attributed to N-H stretching vibrations) [5,27,28,29,30]. The aforementioned peaks were additionally observed in the spectra of all NPP1 to NPP6 samples. However, several of the pharmaceutical compound’s absorption bands overlap with, and are partially masked by, the intrinsic peaks of the NIPU matrix.

In more details, the neat (N1, N2) and the PP-loaded formulations (NPP1 to NPP6) present absorption signals at 1537 cm^−1^ (N-H bending vibrations), 1730 cm^−1^ (C=O carbonyl of ester groups derived from the structure of adipic acid), 1645 cm^−1^ (C=O carbonyl of urethane groups), 2851 and 2927 cm^−1^ (C-H bonds of alkyl groups), 3417 cm^−1^ (N-H stretching vibrations), as well as a broad band in the range of approximately 3128–3643 cm^−1^ (O-H bond of hydroxyl groups resulting from GC ring opening) [23].

The successful encapsulation of PP within NIPU nanoparticles is primarily supported by the presence of peaks attributed to the C=N imine double bond at 1613 cm^−1^ and the C-N single bond at 1271 cm^−1^. Furthermore, the absence of significant shifts in the characteristic peaks of both the drug and the polymer, as indicated by the aforementioned results, suggests that no strong interactions occur between PP and the carrier in the drug-loaded materials [5].

### 3.3. Thermal Transitions (DSC)

**Figure 5:**
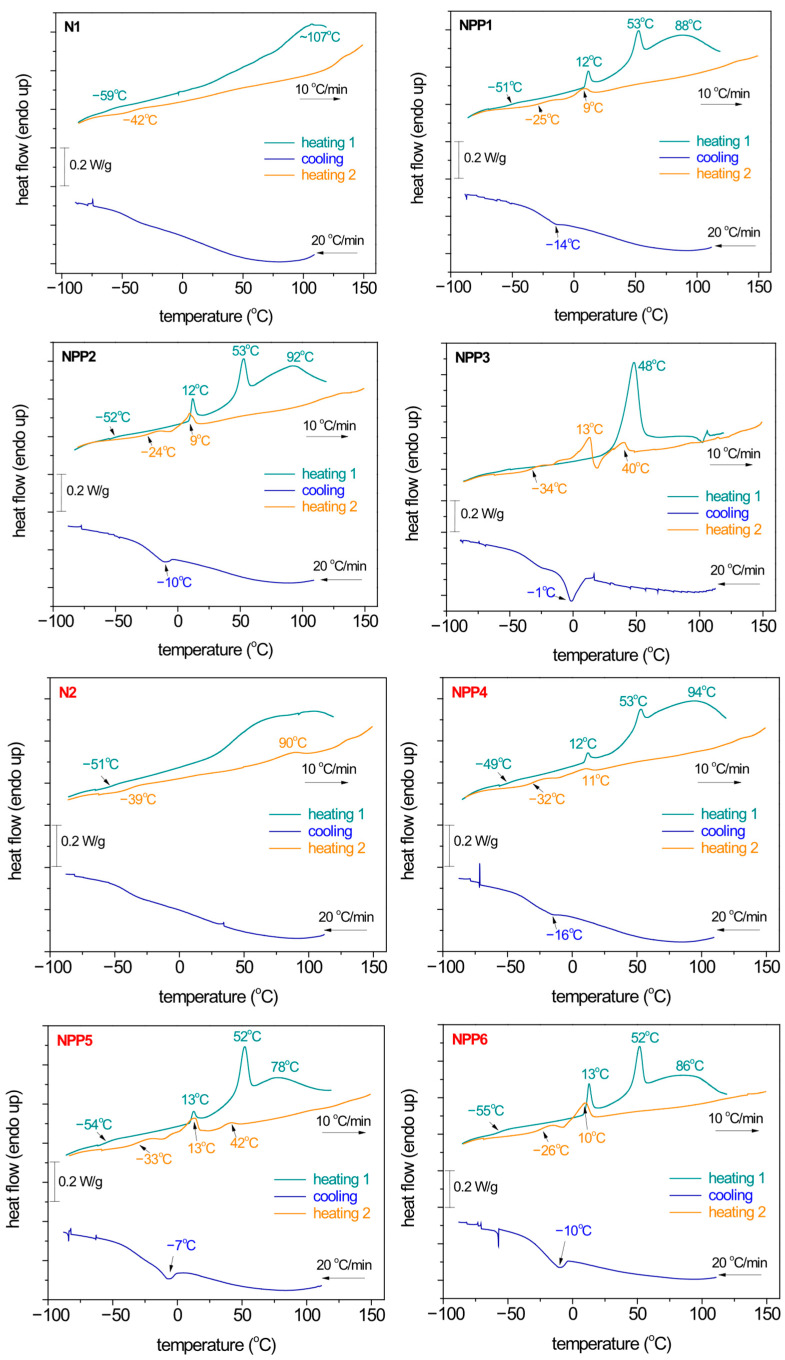
DSC heating/cooling traces for scan 1 and 2 for all samples, indicated on the plots. The shown heat flows have been normalized to the mass of each sample. Indicated are temperatures corresponding to the main thermal events (glass transition, melting, crystallization and solvent evaporation).

Conventional DSC was employed to assess the thermal transitions of the materials, of both the NIPU nanoparticles and the effects of PP. In the first heating run (scan 1), the systems are studied as received, i.e., without any prior thermal treatment nor erasing of the thermal history. During the second run (scan 2), the melted samples were cooled and subsequently heated. The results are shown individually for each sample in Figure 5.

Samples N1 and N2 exhibit clearly amorphous characters, manifested by the recording of glass transition steps at low temperatures, without endothermal/exothermal peaks (melting/crystallization). The characteristic glass transition temperatures (Tg) were determined at the midpoint of the change in heat capacity, ranging from −59 to −39 °C. During the first heating, at high temperatures (50–120 °C), strong and wide exotherms are recorded. These events vanished during the second heating, denoting that the endotherms correspond to irreversible thermal processes, such as evaporation of solvents/humidity/impurities [23]. Upon these irreversible processes, Tg elevates, suggesting a ‘de-plasticization effect’. Comparing between N1 and N2, the latter demonstrates slightly higher Tgs (slightly more rigid material).

It is quite clear, already by a glance at Figure 5, that the presence of PP induces more thermal events, recorded as endothermal and exothermal peaks at T well above the Tg of N1 and N2. Obviously, these are related to the crystallization and melting of PP. The melting peaks are mainly double, being depicted via two individual endotherms, i.e., at 9–14 °C and 40–53 °C during heating. During cooling, the crystallization of PP is recorded as a single exothermal peak between −16 and −1 °C.

**Figure 6:**
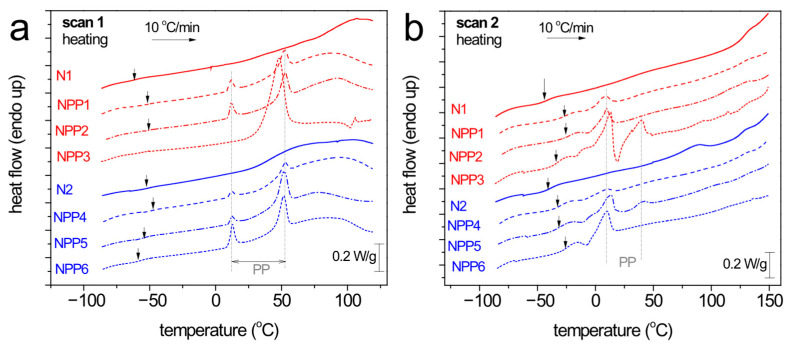
Comparative heating traces for all NIPU-based samples. The heat flow has been normalized to the sample mass, (**a**) scan 1 and (**b**) scan 2. The added arrows mark the position of the ‘glass transition’ steps of NIPU, whereas the vertical dash-dotted lines mark the melting endotherms of PP.

To facilitate a better comparison between the different compositions, we constructed and present Figure 6, which shows heating scans comparatively for the eight samples for scan 1 (Figure 6a) and scan 2 (Figure 6b). Therein, independently from the type of scan, the presence of PP (crystalline) induces, in general, an elevation of the Tg in comparison with the N1 and N2 samples (vertical arrows in the figures). Then, when increasing the PP loading from 0.2 up to 1%, as expected, the strength of the PP’s melting increases systematically. Nevertheless, in scan 1, PP melts at relatively higher temperatures and via two clear and well distinguished peaks. On the other hand, upon the evaporation of the remaining solvents/impurities, in scan 2, the melting of PP occurs at lower temperatures, within a narrower temperature range and by more complex phenomena (cold crystallization, melting, re-crystallization and final melting(s)). An exception to these systematic effects may be the case of NPP3, within which the melting of PP is recorded as a significantly strong single peak at ~48 °C, while at the same time, the glass transition of NIPU seems to vanish.

Table 2 summarizes and compares the temperatures at which the main thermal events of the samples occur (Tg, Tc, Tm).

### 3.4. XRD

**Figure 7:**
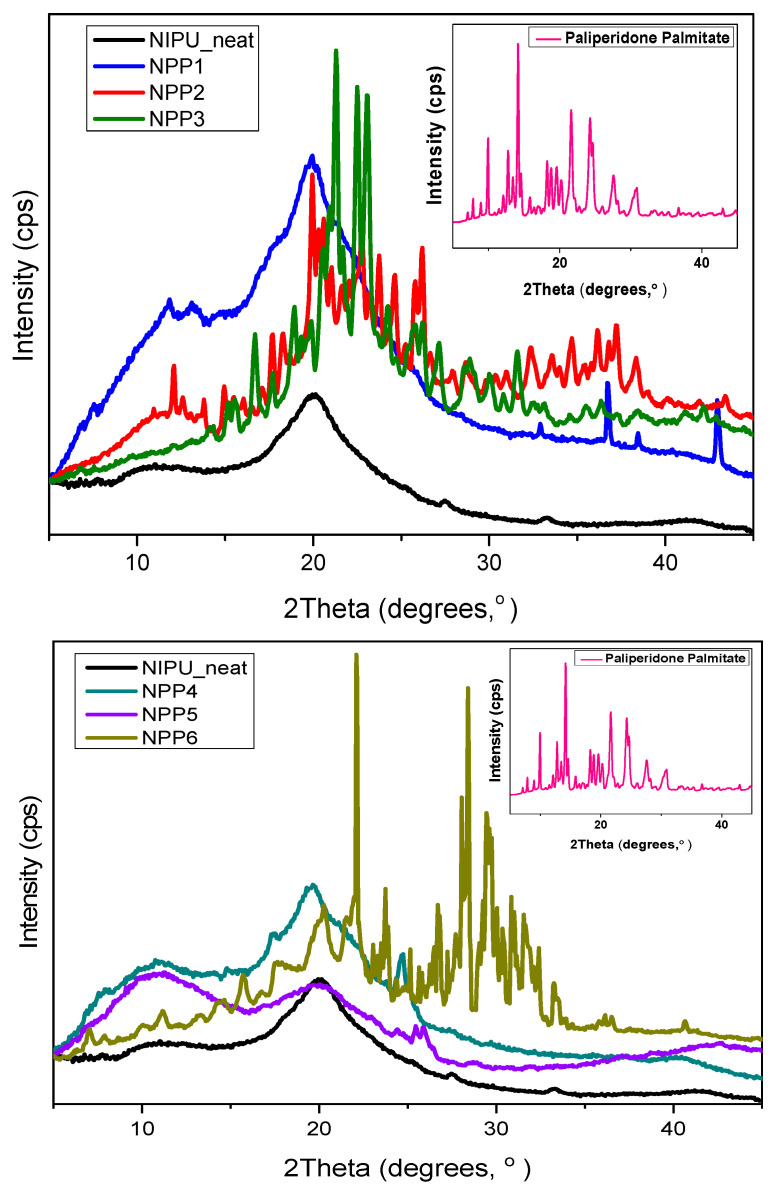
XRD diffractograms of PP, neat NIPU and samples NPP1–NPP6.

The XRD analysis diffractograms of the neat NIPU, PP and NPP1 to NPP6 samples are presented in Figure 7. The multiple sharp peaks observed at the pattern of the drug (at 2θ 9.93°, 14.16°, 18.25°, 21.63°, 24.28°, 24.70°, 27.57° και 30.86°) confirm its crystalline nature, in agreement with the literature [5,31]. In contrast, the broad peaks of the pure NIPU indicate an increased percentage of amorphous regions. From a previous DSC analysis of the neat material [23], the appearance of a melting point implies a semicrystalline nature. Nevertheless, a low crystallinity was recorded, which is consistent with the present XRD results. The absence of prominent sharp peaks can be attributed to the -OH groups of NIPUs which, according to the literature, might hinder the crystallization process, resulting therefore in primarily amorphous materials or exclusively semi-crystalline ones with a limited propensity for microphase separation [32].

The predominant peak in N1, N2 and NPP1–NPP6 samples is recorded at approximately 20° and it aligns with prior published findings on NIPUs [33,34].

By observing the morphology of the diffractograms, it is evident that with the increase in PP in the samples, simultaneously, an increase in the proportion of sharp peaks is detected. While the aforementioned behavior is relatively rare, similar observations have been documented in earlier literature. Some research groups have attributed this phenomenon to the potential plasticizing effect of the drug, which, according to free volume theory, facilitates the mobility of polymer chains and consequently promotes an increase in crystallite number and size [35]. Other studies have suggested that partial recrystallization of the drug within the carrier or surface deposition of drug molecules may provide a plausible explanation [36]. Recrystallization could be associated with the absence of strong interactions between the carrier and the drug [37]—a phenomenon that may also apply to the materials examined in the present study, as no significant associations between the aforementioned components were detected, as evidenced by FTIR analysis.

### 3.5. Mechanical Tests

The mechanical properties reported here correspond to the freeze-dried porous matrices derived from the nanoemulsions and are included as a supplementary characterization of the solid-state material providing insight into the structural integrity during handling and storage, rather than reflecting the mechanical performance of the nanoparticles in suspension. In other words, the measurements are relevant only to the physical stability of the freeze-dried material prior to reconstitution intended for drug delivery and do not directly impact in vivo performance of the nanoparticle system.

The tensile strength analysis of the N1 and N2 samples indicates mechanical properties of a lower magnitude for both materials. In more details, the mean maximum tensile stress of both samples was approximately 0.082 MPa (Table 3). Comparable tensile strength values (0.12 MPa) have been reported in the literature for sponges prepared through lyophilization of single emulsions employing the same PVA concentration (0.5% *w*/*v*) [38]. The primary factor likely responsible for the reduced mechanical strength of the materials are the relatively low concentrations of both PVA and NIPU, which consequently lead to pore walls of decreased density in the final foams and therefore limited mechanical performance. In this context, certain research groups have observed improvements in tensile strength when the concentration of PVA employed was increased [39].

In addition to the above-mentioned factors, several other parameters may have contributed to the observed mechanical strength values, including the low molecular weight of the pure NIPU [40,41], its reduced glass transition temperature (Tg) [42], and the potentially restricted phase separation of the soft and hard segments due to the presence of hydroxyl groups [41], which may prevent the NIPU nanoparticles from acting as a strong reinforcing agent in the sponges.

### 3.6. DLS

**Figure 8:**
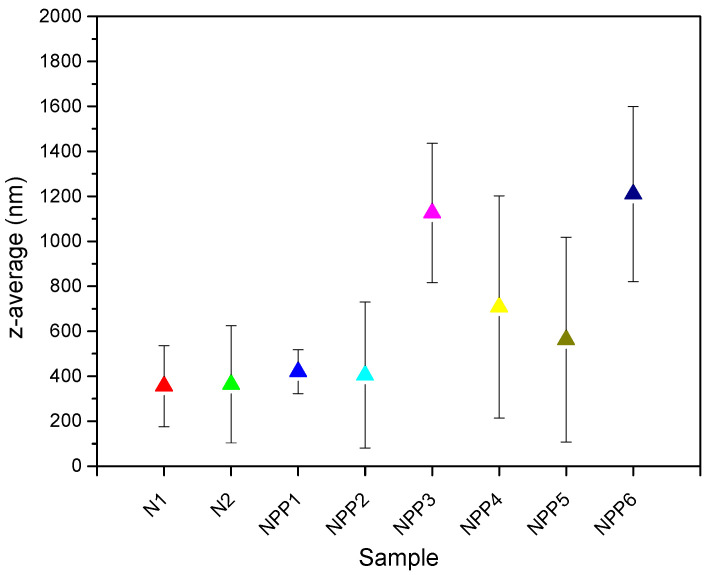
The mean values of the particles’ sizes (z-average size, nm) obtained through DLS analysis.

The average size of the nanoparticles that were obtained via the emulsions was determined using DLS analysis and the results are depicted in Figure 8 and Table 4. It is evident that higher NIPU concentrations yielded nanoparticles of increased size—an observation that is supported by several scientific articles [43,44,45,46,47]. Taghipour et al. [43], Song et al. [46], Budhian et al. [45], as well as Madani et al. [47] all attribute this behavior to the viscosity increase in the organic phase when elevating the polymer concentrations. The higher viscosity renders the disruption of the organic phase’s droplets insufficient when the same power level is applied during emulsification as in formulations with lower polymer amounts.

An additional finding is the increase in nanoparticles’ size with higher amounts of the drug, especially for the concentration of 1%. At PP contents of 0.2% and 0.5% PP, which are closely spaced, the average size of the particles exhibits relatively minimal variation. However, at substantially higher concentrations (1%), the particle size increases markedly. The observation of increasing size with higher drug amounts has also been made by Madani et al. [47], who attribute it to the enhanced availability of the drug within the emulsion droplets or the adsorption of the compound onto the surface of the nanoparticles during the emulsion process.

Furthermore, PDI values of approximately 0.3 were obtained, reflecting a sample with relatively narrow particle-size distribution [48]. In most cases, values exceeding 0.5 are undesirable, as they indicate a broader particle-size distribution [49].

### 3.7. SEM

**Figure 9:**
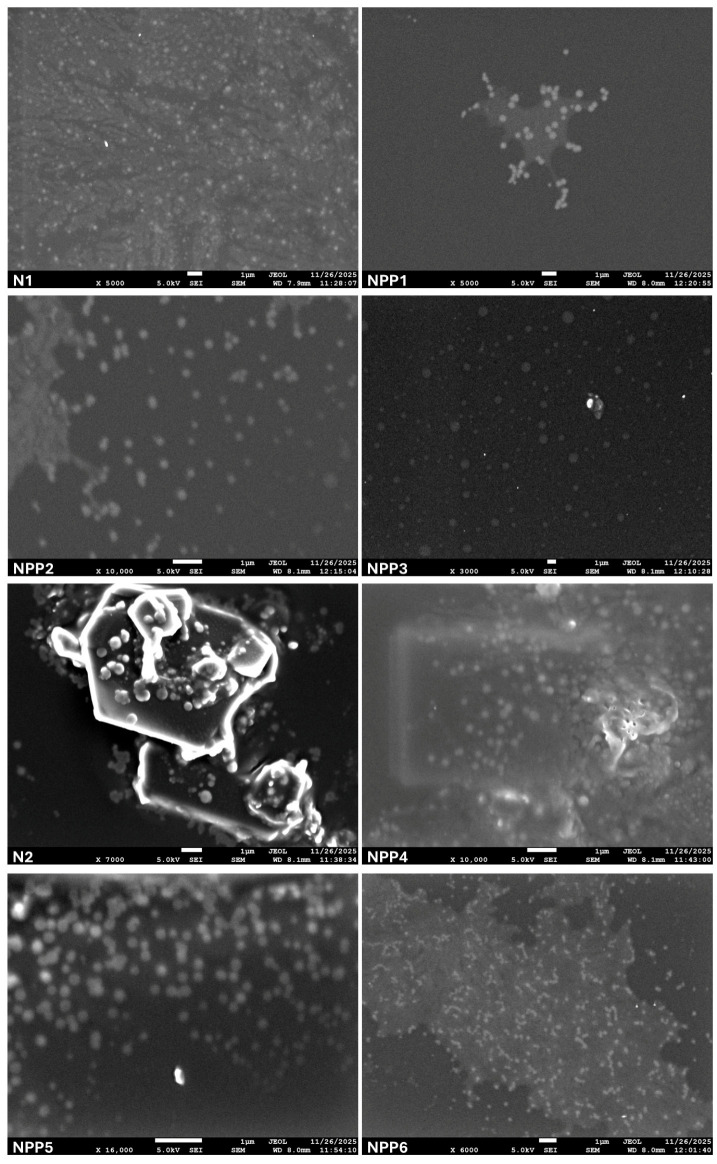
SEM images of non-loaded (N1, N2) and drug-loaded (NPP1–NPP6) nanoparticles.

SEM analysis was performed on N1, N2 and NPP1 to NPP6 samples, targeting the observation of the formed nanoparticles. SEM images of the blank nanoparticles are presented in Figure 9 where a wide dispersion of the carriers is evident. The 1 μm scale bar confirms the reduced size of the particles.

In the SEM analysis of samples NPP1–NPP6 prior to the drug-release study (Figure 9), the nanoparticles are clearly distinguishable and exhibit good dispersion. Furthermore, based on the image scale, their sizes are consistent with the results obtained from DLS analysis.

Nevertheless, certain SEM images depict the partial presence of aggregates, alongside clearly discernible nanoparticles. The aforementioned morphology may be attributed to aggregation occurring during the emulsification process, as primary sonication parameters (amplitude, duration and number of cycles) have been reported to influence or even induce nanoparticle aggregation, particularly under conditions of intensified and prolonged cavitation-induced turbulence within the system [50]. Nanoparticle aggregation may also arise from drying artifacts introduced during SEM sample preparation. Solvent evaporation induces capillary forces and mass transport phenomena, which can lead to non-representative particle clustering and distortion of the original dispersion state [51].

### 3.8. Porosity Test

The results of the porosity test are depicted in Table 5. It is observed that higher concentrations of NIPU (NPP4-NPP6 compared to NPP1-NPP3, and N2 compared to N1) resulted in subtly decreased porosity values. The same behavior was recorded when increasing levels of PP were utilized among samples of equivalent polymer concentration (porosity of NPP1 > NPP2 > NPP3 and porosity of NPP4 > NPP5 > NPP6). According to the literature, this phenomenon can be attributed to elevated concentrations of the emulsions’ organic phase, responsible for inducing lower porosity during the formation of the final foams [52,53,54,55,56]. An indicative example is provided by the work of Xu et al. on polyurethanes for porous scaffolds, in which subtly reduced porosity was observed with increasing amounts of the tailored polymer system they utilized [56].

### 3.9. Water Contact Angle

**Figure 10:**
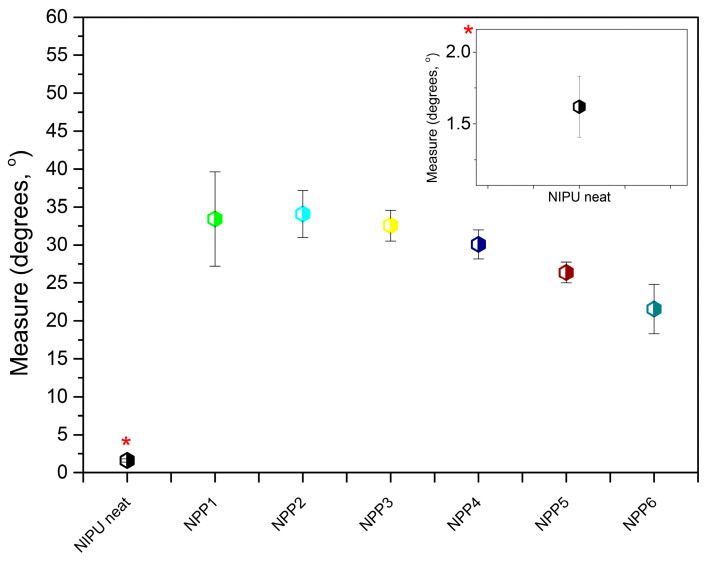
Average change in water contact angle (measure, degrees, °) for the neat NIPU and NPP1–NPP6 samples.

The contact angle measurement was performed on the NPP1–NPP6 samples as well as the neat NIPU. The average initial and final angles of the water droplets were calculated, as well as their average difference (Δθ°/measure,°) which is the y axis of the plot in Figure 10. Higher Δθ/measure values suggest larger deviation between the initial and the final angle, therefore implying that the water droplet expanded further on the sample during the set measurement time. With reference to the theoretical basis of the contact angle [57], for this behavior to occur, the sample must exhibit moderately increased hydrophilicity. Accordingly, the reverse scenario (lower Δθ/measure values and therefore limited angular variation between the initial and the final measurement) corresponds to samples with relatively decreased hydrophilicity/greater hydrophobicity.

From the results presented in Figure 10 and Table 6, it is evident that the average Δθ/measure was the lowest for the neat NIPU, indicating that the pure material possesses the most pronounced hydrophobic properties among the tested samples. In contrast, the drug-loaded emulsion-based foams exhibited higher values relative to the pure material. This behavior is expected considering that, according to the literature, nanoparticle formation through emulsification and freeze-drying is commonly utilized in order to achieve elevated water solubility of hydrophobic moieties, such as pharmaceutical compounds [58]. Furthermore, within the group of NPP1–NPP6 samples, the average Δθ/measure decreased with increasing concentrations of NIPU. These observations are consistent with the aforementioned theoretical framework, since the neat NIPU exhibits hydrophobic characteristics. Likewise, as the proportion of the hydrophobic drug [4] increases, the average Δθ/measure generally exhibits a decreasing trend. Sample NPP2 is the only exception with a value comparable, though slightly higher, than that of the NPP1 sample. Considering the proximity of the drug content in these two samples, the distinct behavior of NPP2 could reasonably be ascribed to the effect of surface roughness on the measured droplet contact angle, particularly since the specimens were not ideally planar [59,60].

### 3.10. Hydrolysis and Enzymatic Hydrolysis

**Figure 11:**
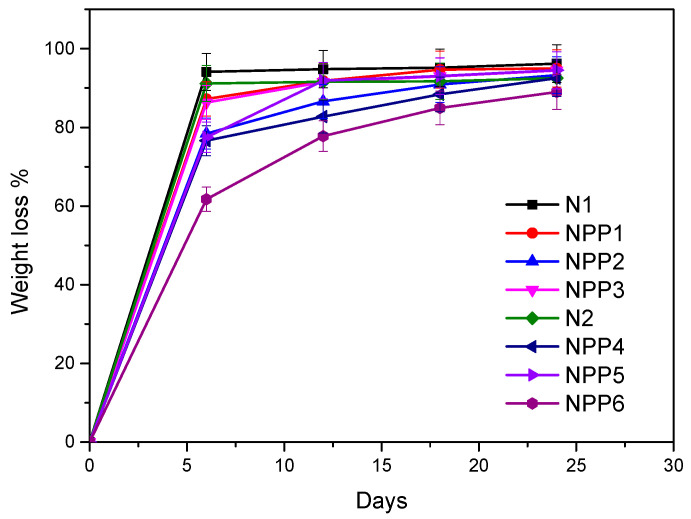
Weight loss (%)—days diagram of samples’ hydrolysis.

**Figure 12:**
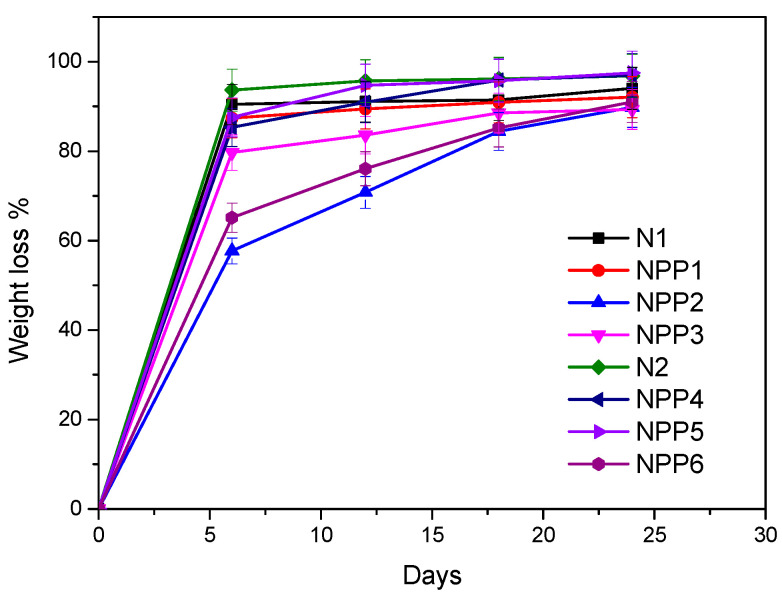
Weight loss (%)—days diagram of samples’ enzymatic hydrolysis.

The hydrolytic and enzymatic degradation of the neat and the drug-loaded samples were performed over a period of 30 days. The weight loss (%) measurements are presented in Figure 11 for hydrolysis and in Figure 12 for enzymatic hydrolysis. It is observed that the unloaded materials, N1 and N2, degraded faster and at a higher percentage than all the drug-loaded samples. Their weight loss (%) during the first measurement (day 6) was the highest compared to the rest of the specimens on both occasions, hydrolysis (94.118% for N1 and 91.206 for N2%) and enzymatic hydrolysis (90.452% for N1 and 93.654% for N2). Generally, the faster/higher degradation of non-loaded samples compared to drug-loaded ones has been widely documented in the literature, especially for hydrophobic drugs. In more details, according to the literature, hydrophobic pharmaceutical agents induce slower/restricted degradation rates by inhibiting water diffusion inside the polymer matrix [61,62,63].

The highest weight difference for all the materials was detected between day 0 and day 6. Thereafter, the differences in weight became less pronounced, and for many samples the final two measurements were closely comparable. On the final day of measurement, the weight loss of the samples ranged from 89.016% to 96.218% under hydrolytic conditions and from 89.815% to 97.502% under enzymatic conditions. These results indicate an extensive material degradation over the course of one month, with minimal remaining mass (<11% of the initial quantity) during the last days, confirming the samples’ biodegradability.

Furthermore, it is evident that for some samples, enzymatic hydrolysis resulted in higher mass loss than hydrolysis without enzymes, while other samples exhibited the exact opposite behavior. A plausible explanation could rely on the enzymes utilized, *Rhizopus arrhizus* and *Pseudomonas cepacia* lipases, which predominately hydrolyze ester bonds [64,65], while the materials of the present work include almost exclusively urethane bonds.

### 3.11. Drug Loading

The drug-loading values of each sample (NPP1–NPP6) are depicted in Table 7.

All formulations exhibited an enhanced capacity for PP encapsulation, considering that drug loadings above 10% are considered high for nanoparticle systems according to the literature [66]. Furthermore, within the NPP1-NPP3 sample series, the initial incorporation of higher PP amounts into the formulation system resulted in increased drug loading within the final polymeric nanoparticles, a finding that is consistent with previous reports [67,68].

The absence of this trend in the NPP4-NPP6 series could be attributed to the influence of nanoparticle size on drug loading, as formulations exhibiting the largest particle sizes in DLS analysis (NPP3, NPP4, NPP6) also demonstrated the highest loading values. This observation has likewise been reported by other scientific groups [69].

The exceptionally high PP loadings suggest that NIPU nanoparticles represent a considerably promising carrier system in terms of the payload capacity of the aforementioned drug. However, we need to acknowledge that the values reflect the mass fraction of PP in the recovered dried formulation and do not necessarily indicate homogeneous molecular dispersion within the polymer matrix, but are likely the result of a combination of polymer-incorporated drug and drug-rich regions within or at the surface of the particles (as evidenced from XRD analysis, showing increased crystallinity with higher drug content).

### 3.12. In Vitro Dissolution

The release profiles of PP from the prepared formulations demonstrate formulation-dependent drug biphasic release behavior over a 14-day period. In all cases, an initial release phase was observed, followed by a slower and more gradual release phase, consistent with diffusion-controlled drug-delivery systems. However, the magnitude of the initial burst and the relative contribution of the slower release phase varied significantly among formulations, whereas not all samples fulfilled the criteria typically associated with sustained-release systems [70,71,72]. Furthermore, to better interpret the release behavior, it is important to analyze it in accordance with the degradation results. As shown in the hydrolysis study, all materials undergo extensive mass loss within the first 6 days, indicating rapid polymer degradation. This behavior is consistent with the release profiles, where the majority of drug release occurs during the early phase of the experiment, followed by minimal additional release thereafter. Therefore, drug release in this system is closely coupled with polymer degradation, suggesting a predominantly degradation-controlled release mechanism, rather than prolonged diffusion from an intact matrix.

**Figure 13:**
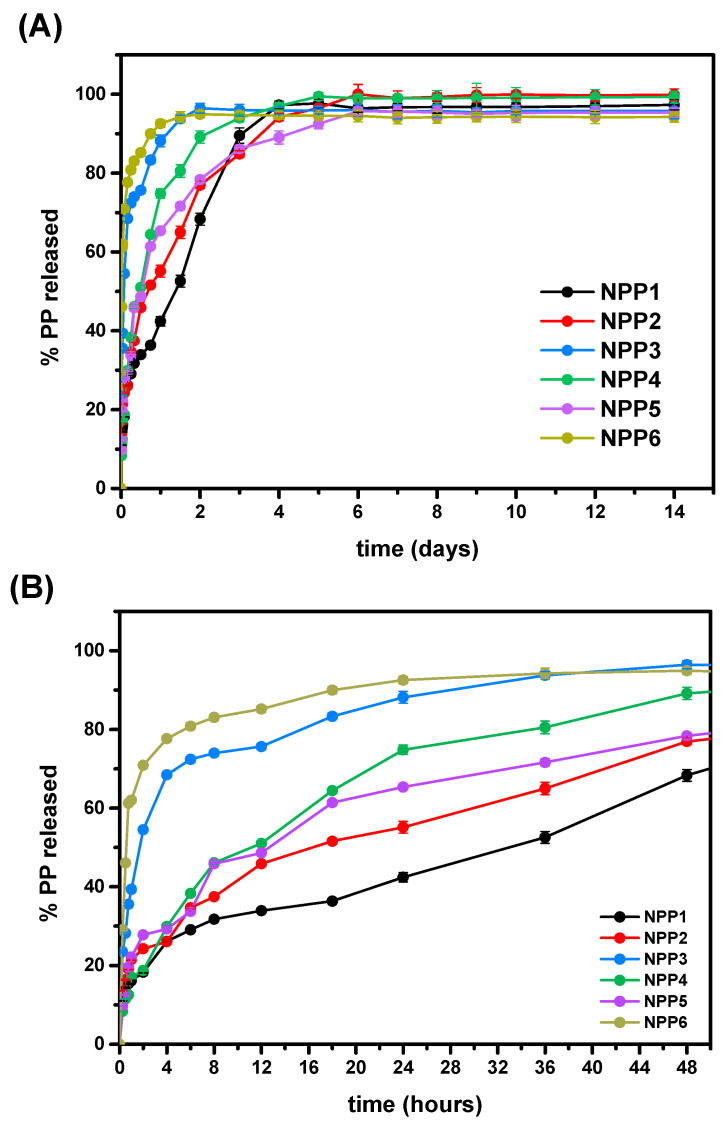
The drug-release profile from NIPU nanoparticles of each formulation during the 14-day period (**A**) and the initial burst release during the first 48 h (**B**).

More precisely, samples NPP1, NPP2, NPP4, and NPP5 exhibited controlled release profiles, characterized by a controlled initial burst and gradual, continuous drug release throughout the 14-day study period (Figure 13A). In contrast, NPP3 and NPP6 displayed excessively rapid drug release during the initial phase, with more than 70% of the encapsulated PP released within the first 12 h (Figure 13B). Such pronounced burst release is inconsistent with sustained-release behavior and suggests that these two formulations may be less suitable for applications requiring controlled or prolonged release of PP. Moreover, the maximum cumulative PP release ranged from 94.94% to 100% across all formulations. The enhanced dissolution rates observed for all formulations suggest improved solubilization of the hydrophobic drug and potentially increased bioavailability [73].

A more detailed analysis of the initial release phase (Figure 13B) reveals formulation-dependent burst behavior. As already stated, samples with notably high drug loadings, NPP3 and NPP6, exhibited the most pronounced burst release, exceeding 70% within the first 12 h. Within the 0.3% NIPU series (NPP1–NPP3), NPP1 displayed a reduced burst release compared to NPP2 and NPP3. Similarly, among the 0.5% NIPU formulations (NPP4–NPP6), NPP5 exhibited the lowest burst effect.

According to previous studies and published work, drug-release kinetics, particularly the burst phase, are known to be influenced by multiple interdependent parameters, including particle size, polymer molecular weight and concentration, porosity, drug loading, and drug distribution within the polymeric matrix. While polymer molecular weight was constant across all samples, polymer concentration, porosity, particle size, and drug loading varied and therefore contributed to the observed differences in release behavior. In general, it is stated that smaller-sized particles tend to exhibit higher burst release due to the smaller diffusion paths that the drug molecules need to cover in order to exit the polymeric carrier [74]. Other approaches include the larger surface-to-volume ratio of smaller particles that contributes towards the enhancement of the drug’s diffusion and release into the medium [75], whereas an additional important factor that could impact the drug-loaded systems is the polymer concentration, since, as previously reported, an increasing polymer concentration results in decreasing drug-release rates, due to bigger polymer matrices and therefore longer diffusion paths [76]. Regarding the porosity parameter, the literature reports that increased burst release is detected in specimens with high porosity values [77], as a result of the larger surface areas and shorter diffusion distances [74], and finally, the drug loading can directly affect the burst release phase, as it is often stated that the burst effect is more prominent for samples that have elevated loadings of the active compound in the polymeric systems [77,78,79].

Within the 0.3% NIPU series, a clear correlation between drug loading and burst release was identified. NPP3, which exhibited the highest drug loading, showed the most pronounced burst release, followed by NPP2, while NPP1 displayed the lowest burst effect. Additionally, particle size analysis revealed that NPP1 contained marginally larger particles than NPP2, which may have contributed to its reduced burst release, as shorter diffusion pathways in smaller particles generally promote faster drug release. Considering both particle size and burst release behavior, NPP1 and NPP2 emerge as the most promising candidates within this series for sustained PP delivery. In the 0.5% NIPU series, no uniform trend was observed across all samples. However, drug loading and porosity appeared to influence burst release behavior between NPP4 and NPP5. In more details, the higher porosity of NPP4 likely facilitated an increased surface area and shorter diffusion distances, therefore leading to a faster initial drug release. The exceptionally high burst release observed for NPP6 is most plausibly attributed to its substantially high drug loading. Consequently, NPP4 and NPP5 represent the most favorable candidates within the 0.5% NIPU series. Comparison of the most promising formulations overall (NPP1, NPP2, NPP4, and NPP5) indicates that NPP1 and NPP2 provide the optimal balance between reduced particle size, relatively high drug loading, and minimized initial burst release. In contrast, NPP3 and NPP6, which exhibited excessive burst release within a short time frame, may be less suitable for applications requiring controlled and prolonged drug delivery.

Interestingly, several observations deviate from commonly reported trends. The formulations exhibiting the highest burst release (NPP3 and NPP6) were also among the largest in particle size and exhibited reduced porosity, contrary to expectations based on diffusion-controlled release mechanisms. These findings suggest that drug loading acted as the dominant parameter governing burst release in this system, outweighing the effects of particle size, porosity, and polymer concentration, and indicating a system-specific release behavior.

A potential mechanistic explanation involves preferential localization of drug molecules near the particle surface, leading to rapid initial release [80]. Such behavior has been associated with solvent evaporation processes, particularly when dichloromethane (DCM) is used and the drug exhibits solubility in the organic phase, promoting drug migration toward the particle surface during solvent removal [74]. Additionally, reduced drug–polymer compatibility may have contributed to the enhanced burst release. XRD analysis revealed high crystallinity of neat PP compared to the predominantly amorphous NIPU matrix, with increasing drug loading leading to higher crystallinity in the final formulations. This increase in crystalline domains, originating primarily from the drug, may have reduced drug–polymer miscibility and amplified burst release behavior [37,81]. Regardless, future investigations, including additional analyses and experiments, will be required to clarify the mechanisms responsible for the behavior observed in the studied samples and their discrepancy with previously reported trends in the literature.

In conclusion, the NIPU-based nanoparticles demonstrated the capacity to provide extended drug release over 14 days, although the extent of sustained behavior is formulation-specific. Drug loading was identified as the primary determinant of the initial burst phase, surpassing the influence of particle size, porosity, and polymer concentration. Based on the balance between minimized burst release and prolonged diffusion-controlled release, NPP1 and NPP2 represent the most promising candidates for the development of sustained injectable PP formulations.

### 3.13. Modeling of Drug Release Profiles

An attempt to fit the experimental release data will be made in the following section. There are several models in the literature serving this purpose. The proposed models are partially empirical and partially phenomenological. Some of them are directly related to physical mechanism of release such as diffusion of the drug in the polymer matrix or erosion of the polymer matrix. There are several recent works reviewing the existing models [82,83]. To refer some of them: It is the zero-order model associated with non-Fickian diffusion, the first-order model associated with Fickian diffusion (for large release amounts), the Higuchi equation associated with the Fickian diffusion from slap-shaped polymer particles and a small released amount [84] (the term Fickian will be omitted hereafter), the Hixson–Crowel model associated with erosion of spherical-shaped polymer particles, the square root of a mass model associated with erosion of cylindrical-shaped particles, and the three-second root of a mass model. The above models are single-parameter ones (not taking into account the asymptotic release as an additional parameter). There are also two parameter models with additional flexibility like the Weibull equation and the Korsmeyer–Peppas model which is a generalization of the Higuchi model. A more physically based parameter model is the series solution of the diffusion equation in a coordinate system relative to polymer particle shape [84]. The above models are in many cases used as empirical ones for a release mechanism different from the one of their derivation. Combinations of the above models are also used in the literature. For example, in [85], a very fast first-order release characteristic of diffusion is combined with an S-shaped (logistic) function characteristic for polymer erosion. Finally, in [86], the Weibull equation is modified in order to coincide with both small- and large-time asymptotes of a diffusion equation. The release of the drug in the present case is considered as a process that occurs through a diffusion mechanism. The data reveals a fast initial burst which is followed by a slower increase in the release up to a saturation value.

**Figure 14:**
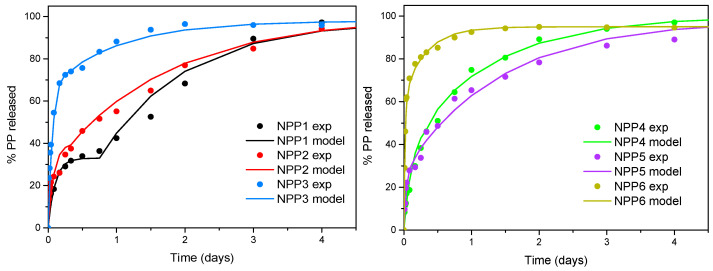
Comparison between experimental release data (symbols) and model (continuous lines).

The initial burst is typically due to the diffusion phenomenon. However, the relevant series solution of diffusion cannot predict the strength of the burst of the present data. Also, the standard empirical models described above do not work appropriately with the present data. This is due to the fact that these are single-mode models, whereas the data appear to obey a dual-mode behavior. A combined-terms model seems to be more effective. The choice here is the sum of two exponentials in the spirit of the combined solution of [85]. Two exponentials are considered. The first is a very fast exponential as in [85]. The second is a slower exponential with a time delay. The mathematical form of the proposed model is:

(3) $$R=R_{1}(1-\exp(-k_{1}t))+R_{2}(1-\exp(-k_{2}(t-t_{m})))$$

where R is the percentage release, R_1_, R_2_ are the percentage fractions of the drug in states 1 and 2 respectively, k_1_, k_2_ are the kinetic constants for the two states and t_m_ is the delay time for state 2. The physical support of the above model is that the drug can be found in two states regarding its diffusion strength. The fast one corresponds to fast diffusion (as in [85]) and the second one to slow diffusion. The delay of the second state can be explained by the lack of this type of drug at the periphery of the polymer particle. The above function is fitted to the experimental data for the six polymer formulations. The comparison between the data and model is presented in Figure 14. The time axis is restricted to 4 days, since most of the information on release kinetics is included in this period. The fitting parameter values for the six formulations are given in Table 8.

It is noted that a small portion of the drug (equal to 100-R_1_-R_2_) remains immobilized in the polymer matrix. The delay time of the second state is decreased as the index of the formulation increases. The second state has an at least one order of magnitude smaller kinetic coefficient that the first one. The fraction of the drug in the second state is larger in formulations NPP1, NPP2 and NPP5, leading to better release characteristics.

## 4. Conclusions

In this study, NIPU nanoparticles encapsulating Paliperidone Palmitate (PP), a hydrophobic antipsychotic used in the treatment of schizophrenia, were developed with the aim of achieving controlled and sustained drug release. Following the synthesis of NIPU via the polyaddition path utilizing adipic acid, glycerol carbonate and 1,6-hexamethylenediamine, oil-in-water (O/W) emulsions were formulated with NIPU concentrations of 0.3% and 0.5% *w/v* in DCM and PVA at 0.5% *w/v* in distilled water. The emulsification process, followed by solvent evaporation and freeze-drying, resulted in the formation of blank nanocarriers, while the incorporation of PP at concentrations of 0.2%, 0.5% and 1% *w/v* into the organic phase of the emulsion enabled the drug’s encapsulation in the polymeric nanoparticle system (samples NPP1–NPP6). FTIR spectroscopy indicated the absence of significant carrier-drug interactions, while DSC and XRD analyses provided the thermal and structural profiles of the formulations. In both heating scans, samples N1 and N2 exhibited glass transition temperatures (Tg) ranging from −59 to −39 °C, and the absence of endothermic and exothermic peaks in N1 confirmed its amorphous nature, in contrast to the semicrystalline behavior of N2, which displayed a melting peak at 90 °C. For samples NPP1–NPP6, Tg values ranged from −55 to −24 °C, melting temperatures (Tm) from 11 to 53 °C, and crystallization temperatures (Tc) from −16 to −1 °C. The observed increase in crystallinity with increasing drug content further supports the absence of strong interactions between NIPU and PP. The sizes of the nanocarriers were between 420 and 1210 nm by DLS and were corroborated by SEM imaging. Mechanical tests of the neat emulsion sponges reveal low tensile strength, while the porosity measurements on both blank and drug-loaded samples indicate a decrease in % porosity with increasing NIPU and PP content. Additionally, drug-loaded formulations exhibited an enhanced hydrophilicity compared to the neat NIPU, as evidenced by water contact angle measurements, where the change in contact angle ranged from 21.6° to 34.1°. Biodegradability was assessed through hydrolysis and enzymatic hydrolysis over a period of one month, revealing minimal residual mass (<11% of the initial amount) in all samples during the final days of the process, thereby confirming their biodegradable nature. Moreover, the drug-loading values of PP in NIPU nanoparticles were particularly high (48.383–77.950%). Finally, the 14-day in vitro release study demonstrated a biphasic delivery profile with a system-specific burst release mechanism. Samples NPP1 and NPP2 were identified as the optimal formulations compared to all drug-loaded specimens, based on their overall characteristics. Regarding the modeling kinetics of drug-release profiles, the release behavior reflects diffusion-controlled kinetics arising from two distinct drug states—an initial burst followed by sustained release. A majority of the formulations showed release profiles consistent with controlled and sustained drug delivery, characterized by a reduced burst effect and extended release duration. The developed NIPU nanoparticles demonstrate several advantages over conventional drug-delivery systems, including high drug-loading capacity, controlled and sustained release profiles with a reduced burst effect, and confirmed biodegradability, which are critical parameters for long-acting formulations. Collectively, these results indicate the suitability of NIPU nanocarriers for the development of injectable formulations for sustained drug release.

## Figures and Tables

**Table 1:** The sample name of each different neat and drug-loaded formulation.

Concentrations of the Components in the Final Produced Formulations	Sample Name
NIPU 0.3%—PVA 0.5%—neat	N1
NIPU 0.5%—PVA 0.5%—neat	Ν2
0.2% PP, NIPU 0.3%—PVA 0.5%	NPP1
0.5% PP, NIPU 0.3%—PVA 0.5%	NPP2
1% PP, NIPU 0.3%—PVA 0.5%	NPP3
0.2% PP, NIPU 0.5%—PVA 0.5%	NPP4
0.5% PP, NIPU 0.5%—PVA 0.5%	NPP5
1% PP, NIPU 0.5%—PVA 0.5%	NPP6

**Table 2:** Comparative summary of thermal transition temperatures for all samples.

Sample	1^st^ Heating Scan		Cooling	2^nd^ Heating Scan	
	Tg (°C)	Tm (°C)	Tc (°C)	Tg (°C)	Tm (°C)
N1	−59	107	-	−42	-
NPP1	−51	12, 53, (88) *	−14	−25	9
NPP2	−52	12, 53, (92) *	−10	−24	9
NPP3	-	48	−1	−34	13, 40
N2	−54	-	-	−39	90
NPP4	−49	12, 53, (94) *	−16	−32	11
NPP5	−54	13, 52, (78) *	−7	−33	13, 42
NPP6	−55	13, 52, (86) *	−10	−26	10

* peaks associated with solvent/humidity evaporation and impurity removal.

**Table 3:** The mean value of the maximum tensile strength of N1 and N2.

Sample Name	Average Max Stress (MPa)
N1	0.08229 ± 0.05
N2	0.08216 ± 0.05

**Table 4:** The z-average (nm) and PDI mean values of the non-loaded and drug-loaded particles.

Sample Name	z-Average (nm)	PDI
**Non-loaded NIPU nanoparticles**		
N1	356 ± 180.10	0.493
N2	364 ± 260.19	0.381
**Nanoparticles loaded with Paliperidone Palmitate**		
NPP1	420 ± 97.61	0.370
NPP2	405 ± 324.40	0.318
NPP3	1126 ± 309.65	0.491
NPP4	708 ± 493.76	0.314
NPP5	563 ± 455.76	0.268
NPP6	1210 ± 389.01	0.248

**Table 5:** The mean porosity (%) of samples N1, N2, NPP1–NPP6.

Sample Name	Porosity (%)
N1	87.37 ± 4.37
N2	86.81 ± 4.24
NPP1	86.53 ± 4.18
NPP2	85.64 ± 4.37
NPP3	81.55 ± 3.91
NPP4	84.75 ± 4.24
NPP5	80.80 ± 3.96
NPP6	79.89 ± 3.95

**Table 6:** Summary table of mean initial and final water contact angles and mean contact angle variation (Δθ) for the neat polymer and samples NPP1–NPP6.

Sample Name	Average Initial θ (°) (t_0_ = 0 s)	Average Final θ (°) (t_f_ = 0.95 s)	Average Δθ (°) (Measure, °)
NIPU neat	56.7	55.1	1.6 ± 0.2
NPP1	52.9	19.5	33.4 ± 6.2
NPP2	48.7	14.6	34.1 ± 3.1
NPP3	55.1	22.5	32.6 ± 2.0
NPP4	54.0	23.9	30.1 ± 1.9
NPP5	69.4	43.0	26.4 ± 1.4
NPP6	60.2	38.6	21.6 ± 3.2

**Table 7:** The % drug-loading values of PP in each formulation.

Sample Name	% Drug Loading
NPP1	56.489 ± 2.82
NPP2	56.905 ± 2.84
NPP3	76.681 ± 3.83
NPP4	77.950 ± 3.9
NPP5	48.383 ± 2.42
NPP6	77.798 ± 3.89

**Table 8:** Parameter found by fitting the model to the experimental data.

Formulation	R_1_	R_2_	k_1_ (1/Days)	k_2_ (1/Days)	t_m_ (Days)
NPP1	33	65	10	0.8	0.75
NPP2	40	60	12	0.6	0.333
NPP3	75	23	14	1	0.333
NPP4	52	48	7	0.8	0.333
NPP5	35	63	20	0.7	0.167
NPP6	70	25	45	3	0.0833

## Data Availability

The original contributions presented in this study are included in the article/Supplementary Material. Further inquiries can be directed to the corresponding author.

## Supplementary Materials

The following supporting information can be downloaded at: https://www.mdpi.com/article/10.3390/polym18080920/s1, Figure S1: NMR spectra of adipic acid, the intermediate product and the produced neat NIPU.
