# Supplementary material for: Sustained Delivery of Paliperidone Palmitate via Encapsulation in Bio-Based NIPU Nanoparticles
Source: Polymers (Basel). 2026 Apr 9;18(8):920. doi: 10.3390/polym18080920 (PMC13120236; doi:10.3390/polym18080920)
Supplement: Supplementary file 1 [file polymers-18-00920-s001.zip › polymers-4212512-supplementary.pdf]

Supplementary material for

Sustained Delivery of Paliperidone Palmitate via Encapsulation in Bio-Based NIPU Nanoparticles

Maria Angeliki Ntrivala [1], Evangelia Balla [1], E.P. Christodoulou [1], Margaritis Kostoglou [2], Panagiotis Klonos [1,3], Apostolos Kyritsis [3] and Dimitrios N. Bikiaris [1,*]

[1] Laboratory of Polymer Chemistry and Technology, Department of Chemistry, Aristotle University of Thessaloniki, GR-541 24 Thessaloniki, Greece; ntrivalamariaangeliki@gmail.com (M.A.N.); evampadio@chem.auth.gr (E.B.); evicius@gmail.com (E.P.C.); pklonos@chem.auth.gr (P.K.)

[2] Laboratory of Chemical and Environmental Technology, Department of Chemistry, Aristotle University of Thessaloniki, GR-541 24 Thessaloniki, Greece, kostoglu@chem.auth.gr (M.K.)

[3] Department of Physics, National Technical University of Athens, Zografou Campus, GR-157 80 Athens, Greece; pklonos@chem.auth.gr (P.K.), akyrits@central.ntua.gr (A.K.)

* Correspondence: dbic@chem.auth.gr; Tel.: +30-231-099-7812

<u>NIPU synthesis process</u>

1 eq. of previously dried A.A. (60 ◦C, vacuum) was added to a round flask along with 1.5 eq. of DMAP, 3 eq. of DCC, and 3 eq. of G.C. The G.C. was added dropwise with a syringe that punctured the septum, which kept the flask closed. The solvent (DCM) was then added dropwise inside the flask until reaching a total volume of approximately 30 mL. The reaction took place under constant stirring in a N_2 atmosphere for 24 h. Because of the DMAP-DCC-DCM employed system, the first step can be classified as a Steglich esterification reaction, allowing the production of the intermediate ester at relatively low temperatures. The verification of the intermediate's successful production was achieved through H-NMR analysis.

After the first step (esterification) the intermediate (ester) was isolated using a vacuum pump and a Buchner funnel. The funnel and the flasks were washed with DCM, and the solvent was collected and supplemented to a final volume of 60 mL. For the intermediate's purification, two-phase extraction with 1M HCl was performed (org:aq 2:1) and the procedure was repeated two times. The obtained purified intermediate was then added to a round flask with 1.5 eq. of 1,6-hexamethylenediamine. The reaction took place at 40 ◦C for 24 h, under stirring and in a N_2 atmosphere. Two-phase extraction was applied again for the purification of the final NIPU, following the same procedure used for the intermediate product. The resulting organic phase was left inside the fume hood for three days and was then transferred to an oven under vacuum for 24 h in order to achieve complete evaporation of the solvent.

<u>NMR analysis of the intermediate product and the neat NIPU</u>

The intermediate product and the neat NIPU were characterized using ^{1}H-NMR spectroscopy to confirm their successful synthesis and verify their structures. In all the spectra, the peak at approximately 2.5 ppm corresponds to DMSO.

The spectrum of the intermediate includes peaks at 1.5–2 ppm, corresponding to H in CH_2 groups. Peaks at 3.25 and 3.9 ppm correspond to H in the groups CH_2-O and CH-O. The success of the first step is primarily evidenced by the absence of peaks at 12 ppm, indicating that there are no OH groups in the intermediate product. This absence suggests that the carboxylic groups of adipic acid reacted efficiently with the hydroxyl groups of glycerol carbonate molecules. The peaks attributed to the CH_2-O and CH-O hydrogens further support the successful intermediate synthesis.

Regarding the neat NIPU's NMR spectrum, the peaks present at 1.25, 1.7, 2.3, 2.9, and 3.25 ppm correspond to the methylene groups' (CH_2) hydrogens. The H in the methine groups (CH) is detected at 3.4 ppm. The peaks at 3.5 and 3.9 ppm are attributed to the H in methylene groups that are next to oxygen via a single bond (CH_2-O). Furthermore, the peak at 5.5 ppm corresponds to the H of the hydroxyl groups (OH), while the peak at 7–8 ppm is attributed to the H in the amino groups that are next to a carbonyl group (NHC=O). The aforementioned data, particularly the peaks attributed to the H in the NHC=O groups, verify the successful synthesis of the NIPU.

The NMR spectrum of the intermediate and the neat NIPU is depicted on Figure S1.

Figure S1

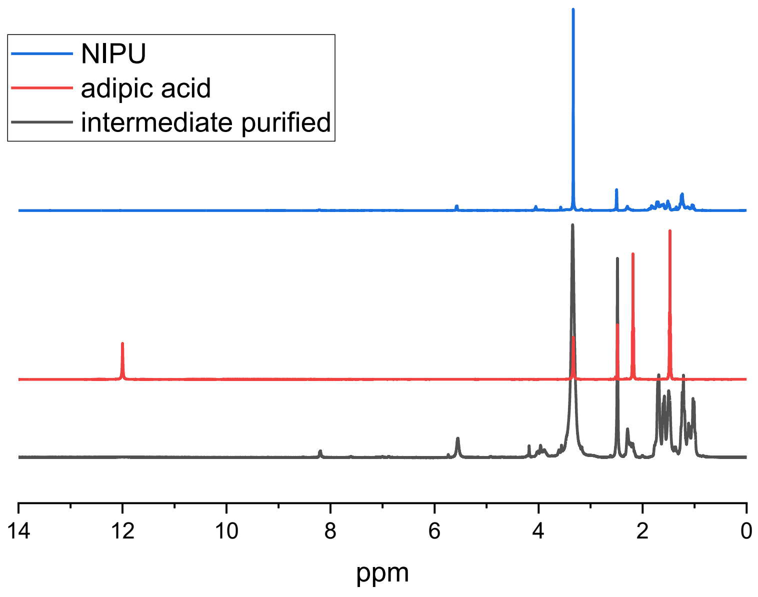

Figure S1. *NMR spectra of adipic acid, the intermediate product and the produced neat NIPU.*
